# Perspectives …

**DOI:** 10.1007/s40037-012-0035-y

**Published:** 2012-11-28

**Authors:** J. van Dalen

**Affiliations:** Skillslab, FHML, Maastricht University, Maastricht, The Netherlands

The success of an educational intervention is dependent on a multitude of factors. Even if we define success as limited as ‘increased competence right after the intervention’, we can safely assume a strong interplay between different aspects of the intervention (for example: time and number of items addressed) and the learning effect caused by that intervention. The problem gets even bigger when we incorporate transfer of learning between different contexts. We often witness that students who have mastered one topic in undergraduate training do not seem to be able to do the same during clerkships.

Among the factors that (co-)influence learning we can assume at least: the content, the format, the didactic skills of the teacher, and the characteristics of the learner.

Regarding content: we have learned that learning (storing new information in our memories, but also retrieving it from our memories) is facilitated when new information is linked to what we know already. Hence the importance of activating prior knowledge when learning new information; this helps to anchor new information *in connection to* what we already know.

We also know to some extent what influence the teaching format has on learning. To generalize: the more active the students are while learning, the higher the learning effect.

And of course the didactic skills of the teacher are important for learning. The teacher can activate students and can help them process, elaborate, and work with what they are learning. Moreover, the teacher’s enthusiasm is known to be a crucial factor in the ease of learning, with a possible mediating role of students’ motivation. Additionally, the teacher is often regarded as a role model, providing an example to the students of how professionals act and react. By the way: in my view that role is generally overestimated. It does not necessarily follow that students will behave like their teacher. Students can identify with a teacher for the time they are dependent on that teacher (for example until they are assessed by that teacher). But in a next stage in their training they can behave in the way the next teacher expects them to, thus showing maximum flexibility in order to survive and pass. I would look forward to reading good studies that attempted to measure the true role modelling as distinguished from ‘survival behaviour’ for the duration of the training.

Learner’s characteristics are another source of influence on learning. Try to compare your own learning to that of others when you try to get used to a new computer programme, or find your way in an unknown holiday environment. Some prefer trial and error; others will first orient themselves until they are sure that no mistake will be made. These issues are addressed in the literature about learning styles, although currently there is much debate about the stability of these ‘styles’ across different situations. Motivation, prior successes, and confidence are important aspects of learners that influence the success of their learning.

In this issue of Perspectives on Medical Education, we proudly present papers addressing all of these viewpoints.

Widyahening et al. [1] report a study into the content and deployment of a course on clinical epidemiology and evidence-based medicine, and its effect in three very different countries: Indonesia, Malaysia, and the Netherlands. They describe the different effects of this course and relate those to the cultural and organizational contexts in which the course was implemented.

Regarding various teaching formats, Gaikwad et al. [2] report a study from India, in which the use of crossword puzzles facilitated the recall of difficult drug names and Balslev [3] gives a description of his PhD work on the use of patient video recording and its effect on learning, on diagnostic accuracy, and the relation of ‘visual expertise’ (looking at the relevant spots) and experience. Prompting new learners to these relevant spots also improved their learning!

In a contribution by six interns from five Dutch medical schools, learning effect and appreciation of a didactic course for medical students is reported [4]. A striking sideline in this paper is that attention for didactics in the medical schools in the Netherlands is low in comparison with other countries. This is striking because the Netherlands have a good reputation in medical education, and because the doctor–patient relationship can benefit strongly from doctors’ didactical competence.

Finally, one important learner characteristic, self-confidence as a result of learning, is addressed in Baldwin’s paper [5]. Junior doctors’ confidence to prescribe or to conduct practical therapies is not high, and undergraduate training is reported as unsatisfactory.

We are proud to be able to share the work of authors in these different fields of health professions education, and we feel that by doing so we do justice to the name of our new journal: Perspectives on Medical Education.
